# Bis(4,4′-sulfanediyldipyridinium) tetra­chloridonickelate(II) dichloride

**DOI:** 10.1107/S1600536812050623

**Published:** 2012-12-15

**Authors:** Julia Werner, Inke Jess, Christian Näther

**Affiliations:** a Institut für Anorganische Chemie, Christian-Albrechts-Universität Kiel, Max-Eyth-Strasse 2, 24118 Kiel, Germany

## Abstract

In the title compound, (C_10_H_10_N_2_S)_2_[NiCl_4_]Cl_2_, the Ni^2+^ cation is tetra­hedrally coordinated by four chloride anions. Two 4,4′-sulfanediyldipyridinium cations and two non-coordinating chloride anions are connected *via* N—H⋯Cl hydrogen-bonding inter­actions into 20-membered rings, in the middle of which are situated the [NiCl_4_]^2−^ complex anions. These rings are stacked in the *b*-axis direction. The Ni^2+^ cation is located on a twofold rotation axis, whereas the chloride anions and the 4,4′-sulfanediyldipyridinium cations occupy general positions.

## Related literature

For background information on this project, see: Boeckmann & Näther (2010 ▶, 2011 ▶); Wöhlert *et al.* (2011 ▶). For the crystal structure of 4,4′-thio­dipyridine, see: Vaganova *et al.* (2004 ▶).
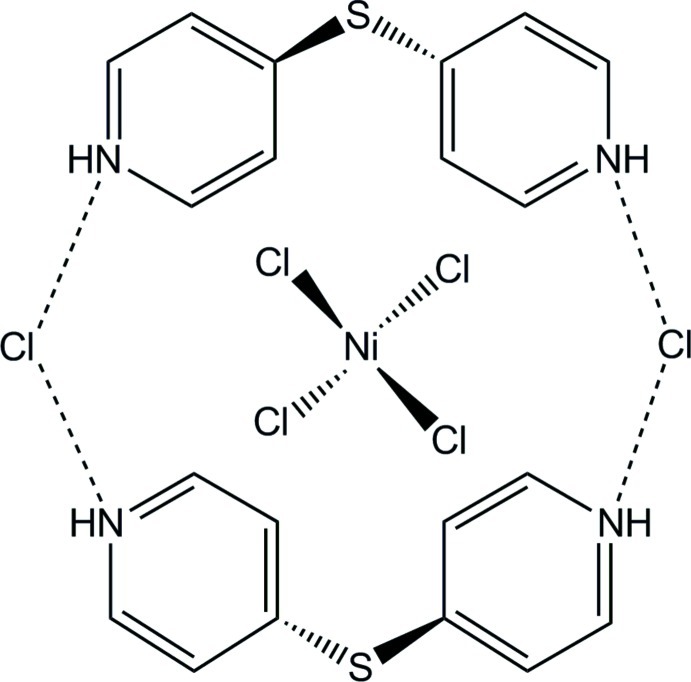


## Experimental

### 

#### Crystal data

(C_10_H_10_N_2_S)_2_[NiCl_4_]Cl_2_
*M* *_r_* = 651.93
Monoclinic, 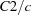

*a* = 19.0497 (9) Å
*b* = 8.0534 (5) Å
*c* = 17.7883 (11) Å
β = 92.368 (6)°
*V* = 2726.7 (3) Å^3^
*Z* = 4
Mo *K*α radiation
μ = 1.47 mm^−1^
*T* = 200 K
0.32 × 0.13 × 0.07 mm

#### Data collection

Stoe IPDS-1 diffractometer
Absorption correction: numerical (*X-SHAPE* and *X-RED32*; Stoe & Cie, 2008 ▶) *T*_min_ = 0.789, *T*_max_ = 0.899
10707 measured reflections
3162 independent reflections
2308 reflections with *I* > 2σ(*I*)
*R*_int_ = 0.062

#### Refinement

*R*[*F* ^2^ > 2σ(*F* ^2^)] = 0.037
*wR*(*F* ^2^) = 0.084
*S* = 0.99
3162 reflections
151 parameters
H-atom parameters constrained
Δρ_max_ = 0.47 e Å^−3^
Δρ_min_ = −0.43 e Å^−3^

### 

Data collection: *X-AREA* (Stoe & Cie, 2008 ▶); cell refinement: *X-AREA*; data reduction: *X-AREA*; program(s) used to solve structure: *SHELXS97* (Sheldrick, 2008 ▶); program(s) used to refine structure: *SHELXL97* (Sheldrick, 2008 ▶); molecular graphics: *XP* in *SHELXTL* (Sheldrick, 2008 ▶) and *DIAMOND* (Brandenburg, 2011 ▶); software used to prepare material for publication: *publCIF* (Westrip, 2010 ▶).

## Supplementary Material

Crystal structure: contains datablock(s) I, global. DOI: 10.1107/S1600536812050623/wm2703sup1.cif

Structure factors: contains datablock(s) I. DOI: 10.1107/S1600536812050623/wm2703Isup2.hkl

Additional supplementary materials:  crystallographic information; 3D view; checkCIF report

## Figures and Tables

**Table 1:** Selected bond lengths (Å)

Ni1—Cl1	2.2569 (7)
Ni1—Cl2	2.2706 (6)

**Table 2:** Hydrogen-bond geometry (Å, °)

*D*—H⋯*A*	*D*—H	H⋯*A*	*D*⋯*A*	*D*—H⋯*A*
N2—H1*N*2⋯Cl3^i^	0.88	2.12	2.987 (3)	168
N1—H1*N*1⋯Cl3	0.88	2.32	3.078 (3)	144

Symmetry code: (i) 
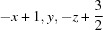
.

**Fig. 1.**
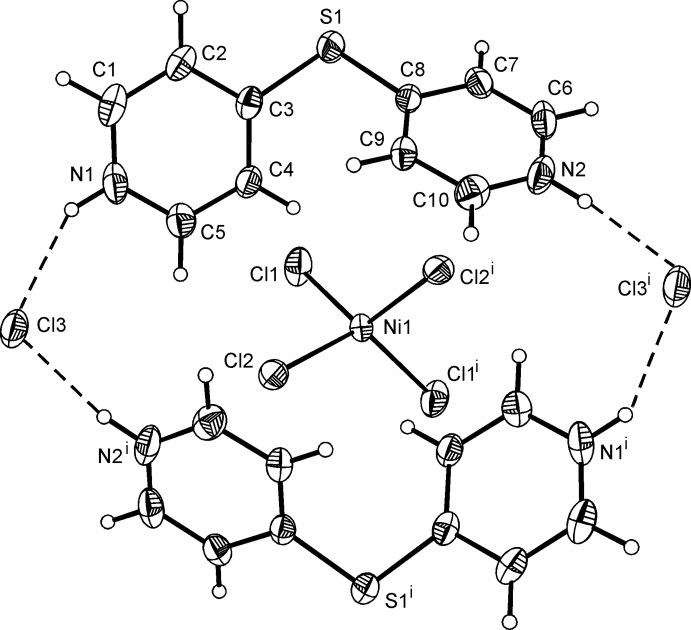
Crystal structure of the title compound with labelling and displacement ellipsoids drawn at the 50% probability level. [Symmetry code: i = -x+1, y, -z+3/2.]

**Fig. 2.**
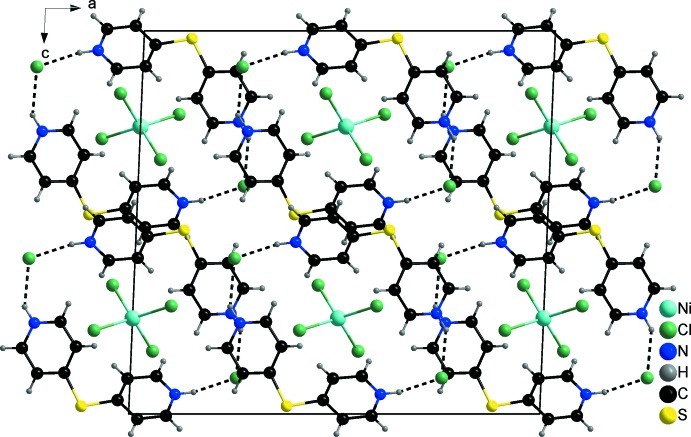
Crystal structure of the title compound in a view along the b-axis. N—H···Cl hydrogen bonding is shown as dashed lines.

## supplementary crystallographic information

### Comment

The title compound was prepared within a project on the synthesis and properties of transition metal thiocyanato coordination polymers (Boeckmann & Näther, 2010, 2011; Wöhlert *et al.*, 2011). During our attempts to prepare a one-dimensional coordination polymer based on 4-chloropyridine as a co-ligand, crystals of the title compound, (C_10_H_10_N_2_S^+^)_2_[NiCl_4_]Cl_2_ (I), have been obtained accidentally and were characterized by single crystal X-ray diffraction.

In the crystal structure of (I) the Ni^2+^ cation is coordinated by four chloride anions within a slightly distorted tetrahedral coordination environment. The complex [NiCl_4_]^2-^ anions are surrounded by two 4,4'-sulfanediyldipyridinium cations and two chloride counter-anions (Fig. 1 and Table 1). Intermolecular N—H···Cl hydrogen bonding is found between the 4,4'-sulfanediyldipyridinium cations and the non-coordinating chloride anions, which leads to the formation of 20-membered rings (Fig. 2 and Table 2). These rings are stacked in the direction of the *b* axis.

The dihedral angle between the pyridine rings in the cations amounts to 52.57 (7) °. The corresponding bond lengths and angles are comparable to those in the neutral 4,4'-thiodipyridine molecule. Slight differences are found with respect to the dihedral angle between the pyridine rings which amounts to 65.4° in the neutral molecule (Vaganova *et al.*, 2004).

### Experimental

Barium thiocyanate trihydrate and 4-chloropyridine hydrochloride were purchased from Alfa Aesar, Ni(SO_4_)_2_^.^6H_2_O was obtained from Merck. Ni(NCS)_2_ was prepared by stirring Ba(NCS)_2_^.^3H_2_O (17.5 g, 56.9 mmol) and NiSO_4_^.^6H_2_O (15.0 g, 57 mmol) in water (500 mL). The white residue of BaSO_4_ was filtered off and the solution evaporated using a rotary evaporator. The homogeneity of the product was investigated by X-ray powder diffraction. The title compound was prepared by the reaction of 26.2 mg Ni(NCS)_2_ (0.15 mmol) and 97.5 mg 4-chloropyridine hydrochloride (0.60 mmol) in 1.5 mL ethanol at 354 K in a closed 10 mL glas culture tube. After one day blue needles of the title compound were obtained. The formation of 4,4'-thiodipyridine starting from 4-chloropyridine in an SCN^-^-containing environment and the presence of free Cl^-^ and complex [NiCl_4_]^2-^ anions seem to be a result of cleavage reactions of both the 4-chloropyridine and SCN^-^ anions. However, the exact mechanism is unclear.

### Refinement

The aromatic H atoms (C- and N-bound) were located in a difference map but were positioned with idealized geometries and were refined isotropically with *U*_iso_(H) = 1.2^.^*U*_eq_(C,N) using a riding model approximation with C—H = 0.95 Å and N—H = 0.88 Å.

### Crystal data

**Table:**

(C_10_H_10_N_2_S)_2_[NiCl_4_]Cl_2_	*F*(000) = 1320
*M**_r_* = 651.93	*D*_x_ = 1.588 Mg m^−^^3^
Monoclinic, *C*2/*c*	Mo *K*α radiation, λ = 0.71073 Å
Hall symbol: -C 2yc	Cell parameters from 10707 reflections
*a* = 19.0497 (9) Å	θ = 2.3–27.9°
*b* = 8.0534 (5) Å	µ = 1.47 mm^−^^1^
*c* = 17.7883 (11) Å	*T* = 200 K
β = 92.368 (6)°	Needle, blue
*V* = 2726.7 (3) Å^3^	0.32 × 0.13 × 0.07 mm
*Z* = 4	

### Data collection

**Table:**

Stoe IPDS-1 diffractometer	3162 independent reflections
Radiation source: fine-focus sealed tube	2308 reflections with *I* > 2σ(*I*)
Graphite monochromator	*R*_int_ = 0.062
Phi scans	θ_max_ = 27.9°, θ_min_ = 2.3°
Absorption correction: numerical (*X-SHAPE* and *X-RED32*; Stoe & Cie, 2008)	*h* = −23→22
*T*_min_ = 0.789, *T*_max_ = 0.899	*k* = −10→10
10707 measured reflections	*l* = −23→23

### Refinement

**Table:**

Refinement on *F*^2^	Secondary atom site location: difference Fourier map
Least-squares matrix: full	Hydrogen site location: inferred from neighbouring sites
*R*[*F*^2^ > 2σ(*F*^2^)] = 0.037	H-atom parameters constrained
*wR*(*F*^2^) = 0.084	*w* = 1/[σ^2^(*F*_o_^2^) + (0.0463*P*)^2^] where *P* = (*F*_o_^2^ + 2*F*_c_^2^)/3
*S* = 0.99	(Δ/σ)_max_ = 0.001
3162 reflections	Δρ_max_ = 0.47 e Å^−^^3^
151 parameters	Δρ_min_ = −0.43 e Å^−^^3^
0 restraints	Extinction correction: *SHELXL*, Fc^*^=kFc[1+0.001xFc^2^λ^3^/sin(2θ)]^-1/4^
Primary atom site location: structure-invariant direct methods	Extinction coefficient: 0.0013 (3)

### Special details

**Table:**

Geometry. All esds (except the esd in the dihedral angle between two l.s. planes) are estimated using the full covariance matrix. The cell esds are taken into account individually in the estimation of esds in distances, angles and torsion angles; correlations between esds in cell parameters are only used when they are defined by crystal symmetry. An approximate (isotropic) treatment of cell esds is used for estimating esds involving l.s. planes.
Refinement. Refinement of F^2^ against ALL reflections. The weighted R-factor wR and goodness of fit S are based on F^2^, conventional R-factors R are based on F, with F set to zero for negative F^2^. The threshold expression of F^2^ > 2sigma(F^2^) is used only for calculating R-factors(gt) etc. and is not relevant to the choice of reflections for refinement. R-factors based on F^2^ are statistically about twice as large as those based on F, and R- factors based on ALL data will be even larger.

### Fractional atomic coordinates and isotropic or equivalent isotropic displacement parameters (Å^2^)

**Table:**

	*x*	*y*	*z*	*U*_iso_*/*U*_eq_	
Ni1	0.5000	0.66427 (5)	0.7500	0.02527 (13)	
Cl1	0.59273 (4)	0.81985 (9)	0.71816 (4)	0.0465 (2)	
Cl2	0.55194 (4)	0.49082 (8)	0.83619 (3)	0.03563 (17)	
Cl3	0.75812 (4)	0.08791 (10)	0.90585 (4)	0.04236 (19)	
N1	0.73877 (13)	0.1738 (3)	0.73764 (14)	0.0425 (6)	
H1N1	0.7637	0.1582	0.7798	0.051*	
C1	0.76591 (16)	0.1281 (4)	0.67229 (18)	0.0424 (7)	
H1	0.8121	0.0839	0.6718	0.051*	
C2	0.72674 (14)	0.1451 (3)	0.60598 (16)	0.0366 (6)	
H2	0.7444	0.1073	0.5598	0.044*	
C3	0.66077 (14)	0.2189 (3)	0.60792 (14)	0.0305 (5)	
C4	0.63496 (14)	0.2699 (3)	0.67604 (14)	0.0319 (5)	
H4	0.5905	0.3227	0.6779	0.038*	
C5	0.67510 (14)	0.2424 (3)	0.74071 (15)	0.0351 (6)	
H5	0.6575	0.2724	0.7880	0.042*	
S1	0.61640 (4)	0.26112 (10)	0.52047 (4)	0.03808 (18)	
N2	0.38935 (13)	0.1530 (3)	0.55137 (14)	0.0394 (5)	
H1N2	0.3443	0.1351	0.5570	0.047*	
C6	0.40903 (15)	0.2520 (4)	0.49540 (15)	0.0383 (6)	
H6	0.3746	0.2986	0.4615	0.046*	
C7	0.47845 (14)	0.2859 (3)	0.48701 (14)	0.0313 (5)	
H7	0.4926	0.3573	0.4479	0.038*	
C8	0.52829 (13)	0.2148 (3)	0.53643 (13)	0.0262 (5)	
C9	0.50641 (14)	0.1074 (3)	0.59261 (14)	0.0284 (5)	
H9	0.5398	0.0541	0.6257	0.034*	
C10	0.43635 (15)	0.0807 (3)	0.59898 (15)	0.0357 (6)	
H10	0.4206	0.0100	0.6376	0.043*	

### Atomic displacement parameters (Å^2^)

**Table:**

	*U*^11^	*U*^22^	*U*^33^	*U*^12^	*U*^13^	*U*^23^
Ni1	0.0281 (3)	0.0202 (2)	0.0271 (2)	0.000	−0.00296 (16)	0.000
Cl1	0.0355 (4)	0.0461 (4)	0.0574 (4)	−0.0070 (3)	−0.0039 (3)	0.0256 (3)
Cl2	0.0370 (4)	0.0359 (3)	0.0341 (3)	0.0052 (3)	0.0038 (2)	0.0137 (3)
Cl3	0.0275 (4)	0.0549 (4)	0.0440 (4)	0.0065 (3)	−0.0057 (3)	−0.0111 (3)
N1	0.0347 (15)	0.0451 (13)	0.0462 (14)	0.0034 (11)	−0.0153 (10)	−0.0021 (11)
C1	0.0290 (17)	0.0392 (16)	0.0586 (19)	0.0058 (11)	−0.0034 (12)	0.0004 (13)
C2	0.0266 (15)	0.0371 (15)	0.0462 (15)	0.0021 (11)	0.0050 (10)	−0.0019 (12)
C3	0.0248 (14)	0.0302 (12)	0.0365 (13)	−0.0030 (9)	−0.0002 (9)	0.0021 (10)
C4	0.0256 (14)	0.0339 (13)	0.0359 (13)	0.0016 (10)	−0.0006 (9)	−0.0040 (10)
C5	0.0351 (16)	0.0326 (13)	0.0372 (14)	0.0001 (11)	−0.0035 (10)	−0.0023 (11)
S1	0.0278 (4)	0.0548 (4)	0.0316 (3)	−0.0045 (3)	0.0014 (2)	0.0041 (3)
N2	0.0240 (13)	0.0422 (13)	0.0520 (14)	−0.0035 (10)	−0.0005 (9)	−0.0154 (11)
C6	0.0339 (16)	0.0375 (14)	0.0421 (15)	0.0076 (12)	−0.0139 (11)	−0.0110 (12)
C7	0.0360 (16)	0.0305 (13)	0.0269 (12)	0.0033 (10)	−0.0051 (9)	−0.0018 (9)
C8	0.0265 (13)	0.0239 (11)	0.0280 (11)	0.0017 (9)	−0.0005 (9)	−0.0082 (9)
C9	0.0282 (14)	0.0236 (11)	0.0330 (12)	0.0000 (9)	−0.0039 (9)	−0.0021 (9)
C10	0.0384 (17)	0.0292 (13)	0.0397 (14)	−0.0060 (11)	0.0038 (11)	−0.0047 (11)

### Geometric parameters (Å, º)

**Table:**

Ni1—Cl1	2.2569 (7)	C4—H4	0.9500
Ni1—Cl1^i^	2.2569 (7)	C5—H5	0.9500
Ni1—Cl2^i^	2.2706 (7)	S1—C8	1.754 (3)
Ni1—Cl2	2.2706 (6)	N2—C10	1.340 (4)
N1—C5	1.336 (4)	N2—C6	1.341 (4)
N1—C1	1.343 (4)	N2—H1N2	0.8800
N1—H1N1	0.8800	C6—C7	1.365 (4)
C1—C2	1.376 (4)	C6—H6	0.9500
C1—H1	0.9500	C7—C8	1.391 (3)
C2—C3	1.392 (4)	C7—H7	0.9500
C2—H2	0.9500	C8—C9	1.398 (3)
C3—C4	1.389 (4)	C9—C10	1.361 (4)
C3—S1	1.772 (3)	C9—H9	0.9500
C4—C5	1.373 (4)	C10—H10	0.9500
			
Cl1—Ni1—Cl1^i^	112.56 (5)	N1—C5—H5	119.7
Cl1—Ni1—Cl2^i^	119.73 (3)	C4—C5—H5	119.7
Cl1^i^—Ni1—Cl2^i^	100.79 (2)	C8—S1—C3	104.00 (12)
Cl1—Ni1—Cl2	100.79 (2)	C10—N2—C6	121.9 (3)
Cl1^i^—Ni1—Cl2	119.73 (3)	C10—N2—H1N2	119.1
Cl2^i^—Ni1—Cl2	104.07 (4)	C6—N2—H1N2	119.1
C5—N1—C1	122.1 (2)	N2—C6—C7	120.1 (2)
C5—N1—H1N1	119.0	N2—C6—H6	119.9
C1—N1—H1N1	119.0	C7—C6—H6	119.9
N1—C1—C2	120.0 (3)	C6—C7—C8	119.2 (3)
N1—C1—H1	120.0	C6—C7—H7	120.4
C2—C1—H1	120.0	C8—C7—H7	120.4
C1—C2—C3	118.7 (3)	C7—C8—C9	119.4 (2)
C1—C2—H2	120.7	C7—C8—S1	116.3 (2)
C3—C2—H2	120.7	C9—C8—S1	124.26 (19)
C4—C3—C2	120.0 (2)	C10—C9—C8	118.6 (2)
C4—C3—S1	122.4 (2)	C10—C9—H9	120.7
C2—C3—S1	117.3 (2)	C8—C9—H9	120.7
C5—C4—C3	118.6 (3)	N2—C10—C9	120.7 (3)
C5—C4—H4	120.7	N2—C10—H10	119.7
C3—C4—H4	120.7	C9—C10—H10	119.7
N1—C5—C4	120.5 (3)		

Symmetry code: (i) −*x*+1, *y*, −*z*+3/2.

### Hydrogen-bond geometry (Å, º)

**Table:**

*D*—H···*A*	*D*—H	H···*A*	*D*···*A*	*D*—H···*A*
N2—H1*N*2···Cl3^i^	0.88	2.12	2.987 (3)	168
N1—H1*N*1···Cl3	0.88	2.32	3.078 (3)	144

Symmetry code: (i) −*x*+1, *y*, −*z*+3/2.
